# Identification of Human Norovirus GII.3 Blockade Antibody Epitopes

**DOI:** 10.3390/v13102058

**Published:** 2021-10-13

**Authors:** Yufang Yi, Shuxia Wang, Xiaoli Wang, Pei Xiong, Qingwei Liu, Chao Zhang, Feifei Yin, Zhong Huang

**Affiliations:** 1 Key Laboratory of Tropical Translational Medicine of Ministry of Education, Hainan Medical University, Haikou 571199, China; yiyufangyyf@163.com; 2 Hainan Medical University—The University of Hong Kong Joint Laboratory of Tropical Infectious Diseases, Hainan Medical University, Haikou 571199, China; 3 CAS Key Laboratory of Molecular Virology & Immunology, Institut Pasteur of Shanghai, Chinese Academy of Sciences, University of Chinese Academy of Sciences, Shanghai 200031, China; sxwang@ips.ac.cn (S.W.); ruociwang@163.com (X.W.); pxiong1@yeah.net (P.X.); qingweiliu2009@163.com (Q.L.)

**Keywords:** norovirus, GII.3 genotype, monoclonal antibody, epitope, histo-blood group antigens, virus-like particle

## Abstract

Human noroviruses are a common pathogen causing acute gastroenteritis worldwide. Among all norovirus genotypes, GII.3 is particularly prevalent in the pediatric population. Here we report the identification of two distinct blockade antibody epitopes on the GII.3 capsid. We generated a panel of monoclonal antibodies (mAbs) from mice immunized with virus-like particle (VLP) of a GII.3 cluster 3 strain. Two of these mAbs, namely 8C7 and 8D1, specifically bound the parental GII.3 VLP but not VLPs of GII.4, GII.17, or GI.1. In addition, 8C7 and 8D1 efficiently blocked GII.3 VLP binding with its ligand, histo-blood group antigens (HBGA). These data demonstrate that 8C7 and 8D1 are GII.3-specific blockade antibodies. By using a series of chimeric VLPs, we mapped the epitopes of 8C7 and 8D1 to residues 385–400 and 401–420 of the VP1 capsid protein, respectively. These two blockade antibody epitopes are highly conserved among GII.3 cluster 3 strains. Structural modeling shows that the 8C7 epitope partially overlaps with the HBGA binding site (HBS) while the 8D1 epitope is spatially adjacent to HBS. These findings may enhance our understanding of the immunology and evolution of GII.3 noroviruses.

## 1. Introduction

Noroviruses (NoVs) are a group of non-enveloped RNA viruses belonging to the *Norovirus* genus in the *Caliciviridae* family, and they are the leading cause of sporadic and epidemic nonbacterial acute gastroenteritis (AGE) in humans [1,2,3]. NoVs possess a single-stranded, positive-sense RNA genome about 7.5 ~ 7.7 kb in length [4,5], which contains three open reading frames (ORF): ORF1 encodes the replicase polyprotein, ORF2 encodes the major capsid protein named VP1, and ORF3 encodes the minor capsid protein named VP2 [4,6,7]. VP1 capsid protein consists of a shell (S) domain and a protruding (P) domain that can be further divided into two subdomains, namely P1 and P2 [4,6,7]. The P2 domain of most NoVs harbors binding sites for human histo-blood group antigens (HBGAs) [8,9,10], which are complex, fucose-containing carbohydrates present abundantly on the intestinal epithelia and function as an attachment receptor for human NoVs [11,12,13].

Based on the VP1 amino acid sequence, NoVs were classified into six genogroups (GI to GVI) in 2013 [14]. This NoV classification scheme was recently updated, with the number of genogroups expanded to 10 (GI to GX) [3,14]. Viruses of GI, GII, and GIV infect humans, and in particular, GII, which comprises 27 genotypes [14], accounts for approximately 90% of norovirus infections in humans [15]. Among all GII genotypes, GII.4 has been the predominant one causing AGE in humans of all ages over the past two decades [16,17,18,19,20], while GII.3 is one of the most common genotypes associated with NoV infection in infants and young children [21,22,23,24,25,26,27,28,29,30]. In particular, one clinical study showed that GII.3 and GII.4 were responsible for 71.24% and 23.53% of NoV-associated pediatric AGE, respectively, in Hohhot, China, between January 2012 and December 2017 [27]. It was estimated that 70% of children would have been infected by GII.3 by 2 years of age [31].

GII.3 NoVs undergo constant evolution, driven primarily by intergenic recombination [21,22,32]. The initial phylogenetic analysis, which was published in 2011, divided GII.3 NoVs into three clusters (I, II, and III) based on the available 63 GII.3 VP1 sequences [21]. Two years later, these relatively larger clusters were further defined into five smaller lineages (A to E), which were generally observed to be temporally sequential in terms of collection dates of the corresponding strains within each lineage [22]. In 2020, Saito et al. performed a phylogenetic analysis of a large number of sequences of GII.3 strains, most of which were collected after 2013, and therefore updated GII.3 classification with the analyzed strains being divided into three clusters (1, 2, and 3) based on the VP1 amino acid sequence [33].

The GII.3 VP1 protein can self-assemble into virus-like particles [34,35,36], with the outer P domain in either “resting” or “rising” conformation depending on the pH of sample solutions [35]. GII.3 VLPs formed by the entire VP1 protein or P particles solely made of the P domain can bind HBGAs in vitro [8,21,34,37]. A recent structural study has defined an HBGA binding site on GII.3, which is constituted by eight VP1 residues within the P2 subdomain [36]. Immunization of animals with GII.3 VLPs elicited antibodies capable of blocking the interaction between HBGAs and homotypic VLPs [34,37], indicating that the GII.3 VP1 protein does contain blockade antibody epitopes. Although in silico analyses have predicted several sites where GII.3 blockade antibody epitopes might reside [21,22,33], thus far, the exact locations of GII.3 blockade antibody epitopes have not been defined experimentally.

In the present study, we generated and characterized four monoclonal antibodies (mAbs) from mice immunized with VLP of a GII.3 cluster 3 strain. Two of these mAbs, designated 8C7 and 8D1, exhibited potent binding and blockade activity toward GII.3 VLP but not VLPs of GII.4, GII.17, or GI.1, demonstrating that 8C7 and 8D1 are GII.3-specific blockade mAbs. By using a series of chimeric VLPs, we mapped the epitopes of 8C7 and 8D1 to residues 385–400 and 401–420 within the P2 subdomain of the VP1 protein, respectively. Both 8C7 and 8D1 epitopes are surface exposed. The 8C7 epitope overlaps with the HBGA binding site (HBS), while the 8D1 epitope is spatially adjacent to HBS. Sequence alignment showed that the two blockade antibody epitopes are highly conserved within the GII.3 cluster 3. Our work thus provides new insight into the immunology and evolution of GII.3 NoVs.

## 2. Materials and Methods

### 2.1. VLP Production

Norovirus GII.3 strains (Table 1), including Hu/GII.3/CUHK-NS-227/2014/CHN (GenBank ID: KJ499444; hereafter called GII.3-KJ), Hu/GII.3/3-34/2015/HNZZ/CHN (ID: KY767665; hereafter called GII.3-KY), Hu/GII.3/RotterdamP8D31/2006/NL (ID: AB385642; hereafter called GII.3-AB38), and Hu/NoV/GII.3/SaitamaU201/1998/JPN (ID: AB067542; hereafter called GII.3-AB06), were chosen for VLP production in PichiaPink™ yeast according to our previously described procedures [38,39]. Briefly, the optimized VP1 gene of each GII.3 strain was cloned into the vector pPink-HC (Invitrogen, Waltham, MA, USA), and the resulting plasmids were transformed into PichiaPink^TM^ Strain 1 (Invitrogen). The resultant yeast transformants were cultured and then induced for protein expression. After lysis and centrifugation of the cells, the supernatant was subjected to polyethylene glycol (PEG) precipitation and sucrose-gradient ultracentrifugation, yielding purified VLP samples. The GI.1, GII.4, and GII.17 VLPs were generated in our previous study using the PichiaPink™ yeast expression system [38]. The purified VLPs were quantified using Bradford assay.

### 2.2. Generation of Anti-GII.3 mAbs

The mouse immunization study was approved by the Institutional Animal Care and Use Committee at the Institut Pasteur of Shanghai (project ID: A2016013; approval date: 21 April 2016).

Female BALB/c mice were injected intraperitoneally with 10 µg of GII.3-KJ VLP plus 500 µg of alum adjuvant (InvivoGen, San Diego, CA, USA) at days 0, 26, and 44. The mice were boosted on day 46 with 10 µg of GII.3-KJ VLP. Three days later, splenocytes isolated from the immunized mice were used to generate hybridomas using our previously described protocols [40,41,42]. The resulting hybridomas were screened for IgG antibodies binding to GII.3 VLP by ELISA as described below. The positive hybridoma clones were chosen for further expansion, and immunoglobulin isotypes were determined using the SBA Clonotyping System-HRP Kit (SouthernBiotech, Birmingham, AL, USA). Hybridoma cells were expanded and then injected intraperitoneally into female Balb/c mice previously irritated with liquid paraffin. Ascitic fluids were then obtained from the mice and clarified by centrifugation. The mAbs were then purified from the supernatants using the HiTrap^TM^ Protein G affinity column (GE Healthcare, Chicago, IL, USA), according to the manufacturer’s instructions. Briefly, Protein G resin was pre-equilibrated with 0.15 M PBS. Ascitic fluids were diluted with PBS, filtered through 0.45 μm Millipore filter, and then loaded onto the column. After extensive wash with PBS, the bound antibody was eluted with 0.1 M glycine-HCl (pH 2.8), and the fractions were collected in tubes containing 1 M Tris-HCl (pH 9.0). The purified mAbs were examined by SDS-PAGE.

### 2.3. VLP-Binding ELISA

ELISA plates (Thermo Fisher Scientific, Waltham, MA, USA) were coated with 50 ng/well of purified VLPs and incubated at 37 °C for 2 h, followed by blocking with 5% non-fat milk diluted in PBST. Then, hybridoma culture supernatants (50 μL/well), anti-GII.3-VLP mouse sera (1/1000 dilution), anti-GII.3 mAbs (50 ng/well), a norovirus cross-reactive mAb 7D8 (50 ng/well) [38], or an irrelevant anti-SARS-CoV-2 mAb 2H2 (50 ng/well) [41] were added to the plates and incubated for 2 h at 37 °C. After washing, the plates were incubated with horseradish peroxidase (HRP)-conjugated secondary antibody for 1 h at 37 °C. After washing, color was developed with the TMB substrate system, and the absorbance was then determined at 450 nm wavelength.

### 2.4. Biolayer Interferometry Assay (BLI)

Prior to BLI assay, purified GII.3-KJ VLP was labeled with biotin using the EZ-Link sulfo-NHS-LC-LC-biotin kit (Thermo Fisher Scientific) and then purified using Zeba™ spin desalting column (Thermo Fisher Scientific). To measure the binding affinity of mAbs to GII.3 VLP, BLI experiments were performed in the Octet^®^ RED96 System (Pall FortéBio, Fremont, CA, USA). Specifically, the streptavidin (SA) biosensors were dipped into the biotinylated GII.3 VLP solution until saturation, followed by rinsing with kinetics buffer. The VLP-coated biosensors were incubated with various concentrations of the mAbs and then dissociated in kinetics buffer. Data were analyzed using Octet data analysis software (version 11.0; Pall FortéBio).

### 2.5. VLP/Pig Gastric Mucin (PGM) Binding Blockade Assay

The ability of the mAbs to inhibit the interaction between GII.3 VLP and HBGA were tested by the VLP/PGM blockade assay according to our previously reported protocol [38]. Briefly, ELISA plates were coated with 500 ng/well of pig gastric mucin (PGM) type III (Shanghai Yuanmu Biotech, Shanghai, China), followed by blocking with 5% milk. MAb samples were serially diluted and mixed with 0.5 µg/mL of VLP. After incubation for 1 h, the VLP/mAb mixtures were added to the plates and incubated for 1 h. After washes, the plates were incubated with a rabbit anti-GII.3 polyclonal antibody for 1 h, followed by incubation with HRP-conjugated anti-rabbit IgG (Sigma-Aldrich, St. Louis, MO, USA). After color development, the absorbance was determined at 450 nm. The 50% inhibition concentration (IC50) was calculated using GraphPad Prism version 8.

### 2.6. Epitope Mapping

A set of chimeric plasmids was constructed by replacing different portions of GII.3-AB38 VP1 with the counterparts from GII.3-KJ VP1. These plasmids were used to express chimeric GII.3 VLPs in PichiaPink™ yeast according to our previously described procedures [38,39].

For epitope mapping, an ELISA assay was performed. Briefly, 100 µL/well of individual VLP-containing yeast lysates were coated onto the plates and incubated at 37 °C for 2 h, followed by blocking with 5% milk/PBST. Then, the plates were incubated with rabbit anti-GII.3 polyclonal antibody (1/3000 dilution; control) or anti-GII.3 mAbs (50 ng/well) for 2 h at 37 °C, followed by incubation with the corresponding HRP-conjugated secondary antibodies. After washing, color was developed, and the absorbance was then determined at 450 nm.

### 2.7. Sequence Alignment

VP1 protein sequences from different norovirus GII.3 strains were aligned using CLC Sequence Viewer software (v6.8).

### 2.8. Structural Representation of mAb Epitopes

The epitopes of mAbs 8C7 and 8D1 and the HBGA binding site were depicted on the GII.3 P domain dimer structure (strain TV-24; PDB: 6IR5) using UCSF ChimeraX software (v1.2.5). Note that the P domain structure of the GII.3 KJ strain, which was used for mAb production, has not been resolved.

## 3. Results

### 3.1. Generation and Characterization of the Anti-GII.3 mAbs

To develop GII.3-specific mAbs, we immunized Balb/c mice with VLP of the GII.3 cluster 3 strain Hu/GII.3/CUHK-NS-227/2014/CHN (GenBank ID: KJ499444; designated as GII.3-KJ, Table 1). Splenocytes from the VLP-immunized mice were fused with SP2/0 myeloma cells to generate hybridomas. Culture supernatants from the resulting hybridomas were analyzed by ELISA for their reactivity with the immunogen VLP. The results showed that four individual hybridoma clones, designated 3A3, 8C7, 8D1, and 9B8, were ELISA-positive (Table 2). Isotyping analysis revealed that all of these four clones were IgG1 antibodies (Table 2).

**Figure 1:**
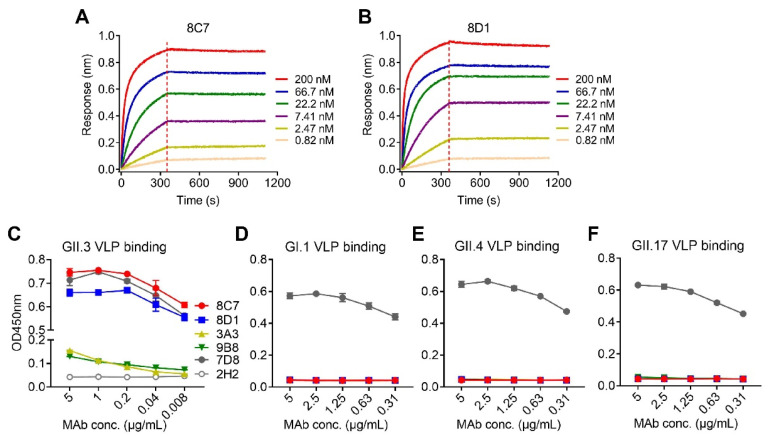
Binding affinity and specificity of the mAbs to GII.3 VLP. (**A**,**B**) Biolayer interferometry sensorgrams of anti-GII.3 mAbs 8C7 (**A**) and 8D1 (**B**) binding to biotinylated GII.3-KJ VLP on streptavidin-coated biosensors. Association and dissociation steps are divided by dashed lines. The mAb concentrations were shown. (**C**–**F**) Reactivity of anti-GII.3 mAbs (8C7, 8D1, 3A3, and 9B8) with GII.3-KJ VLP (**C**), GI.1 VLP (**D**), GII.4 VLP (**E**), and GII.17 VLP (**F**) were determined by ELISA. Data are mean ± SD of triplicate wells. 7D8, a norovirus cross-reactive antibody, used as a positive control. 2H2, an anti-SARS-CoV-2 mAb, used as a negative control. Conc., the abbreviation for concentration.

We performed biolayer interferometry (BLI) assays to determine the binding affinity of the mAbs to the GII.3 VLP. The results showed that 8C7 and 8D1 possessed high affinity to the GII.3 VLP with KD values of <0.001 nM, whereas 3A3 and 9B8 had a much lower affinity with KD values being 31 and 0.161 nM, respectively (Table 2). Representative BLI graphs for 8C7 and 8D1 mAbs are shown in Figure 1A,B.

Then, we assessed the binding specificity of the four mAbs by performing ELISAs with VLPs of four different genotypes (GII.3, GII.4, GII.17, and GI.1) as coating antigen (Figure 1C–F). MAbs 8C7 and 8D1 strongly reacted with the GII.3 VLP in an antibody dose-dependent manner, whereas only high concentrations of 3A3 and 9B8 yield detectable reactivity with the GII.3 VLP (Figure 1C), in consistency with their binding affinity measured by BLI (Table 2 and Figure 1A,B). In contrast, none of the four anti-GII.3 mAbs showed binding activity to the GI.1 VLP, the GII.4 VLP, or the GII.17 VLP, regardless of the antibody concentration (Figure 1D–F). As the positive control, a previously identified norovirus cross-reactive mAb, 7D8, was found to efficiently bind with each of the four VLPs (Figure 1C–F), validating the assays. Based on the above data, we conclude that the four mAbs generated here are indeed GII.3 specific.

### 3.2. Blockade Activity of the Four Anti-GII.3 mAbs

**Figure 2:**
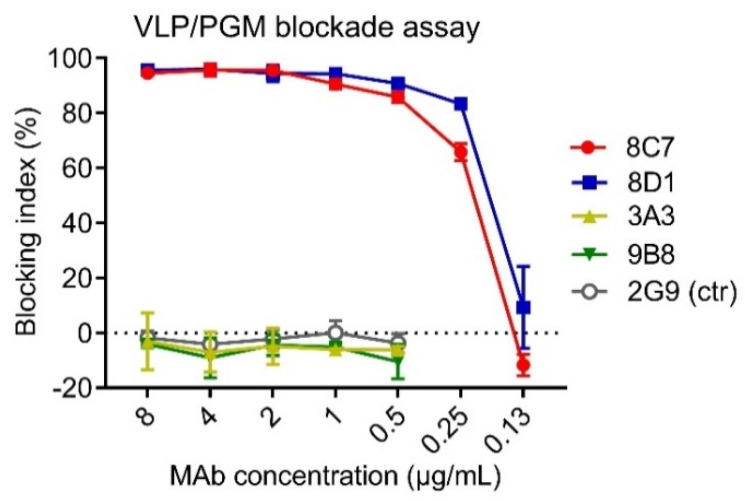
The ability of the mAbs to block the interaction between GII.3-KJ VLP and HBGA-containing PGM III was tested by ELISA. HBsAg-specific mAb 2G9 was used as a control (ctr). Blocking index (%) was determined by comparing the results of the VLP/MAb wells with the VLP-only wells. Data are mean ± SD of triplicate wells.

HBGAs are considered a binding receptor for NoVs [43,44]. Vaccine-elicited antibody titers that blocked VLPs binding with HBGAs correlated with protection in clinical trials [45,46], and hence HBGA blockade ELISAs have been used as a surrogate neutralization assay to assess the efficacy of candidate vaccines and antibodies [47,48]. To evaluate the functions of the four anti-GII.3 mAbs, we performed blockade ELISAs with pig gastric mucin (PGM) type III as the source of HBGAs [49]. As shown in Figure 2, 8C7 and 8D1 mAbs efficiently blocked the immunogen GII.3 VLP binding to PGM with IC50s of 0.24 µg/mL and 0.19 µg/mL, respectively, whereas no blocking effect was observed for 3A3 and 9B8 as well as for the isotype control mAb, regardless of the antibody concentrations used. These data demonstrate that 8C7 and 8D1, but not 3A3 and 9B8, are potent blockade mAbs. Hence, we focused our subsequent analyses on 8C7 and 8D1.

### 3.3. Intratypic Cross-Binding by 8C7 and 8D1 mAbs

**Figure 3:**
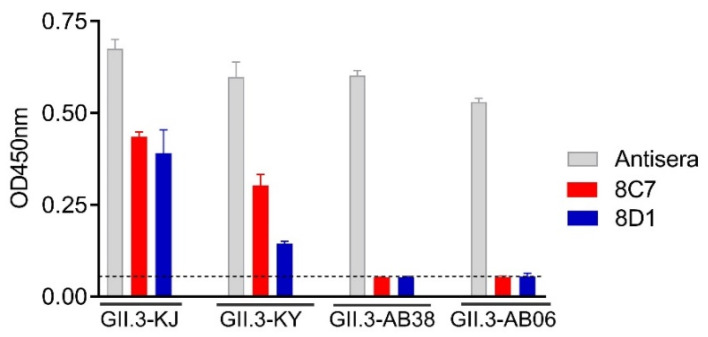
Cross-reactivity of the 8C7 and 8D1 mAbs was tested by ELISA with various wild-type GII.3 VLPs. Rabbit anti-GII.3 VLP antiserum was used as a positive control. Dashed line indicates the value from the blank well (containing no antigen). Data are mean ± SD of triplicate wells.

To examine the mAbs’ cross-reactivity within GII.3 genotype, VLPs of the strains GII.3-KY (cluster 2, according to Saito et al. [33]), GII.3-AB38 (cluster 2), and GII.3-AB06 (cluster 1) were produced (Table 1). These VLPs were compared with the immunogen VLP (GII.3-KJ, cluster 3) by ELISA for their reactivity with 8C7, 8D1, or the rabbit anti-GII.3-KJ VLP sera. As expected, the rabbit anti-GII.3 sera could react with all VLPs, serving as a positive control in this assay (Figure 3). The 8C7 and 8D1 mAbs efficiently bound the original (GII.3-KJ) VLP and the GII.3-KY VLP but did not show significant reactivity to the VLPs of GII.3-AB38 and GII.3-AB06 (Figure 3).

### 3.4. Epitope Mapping for 8C7 and 8D1 mAbs

**Figure 4:**
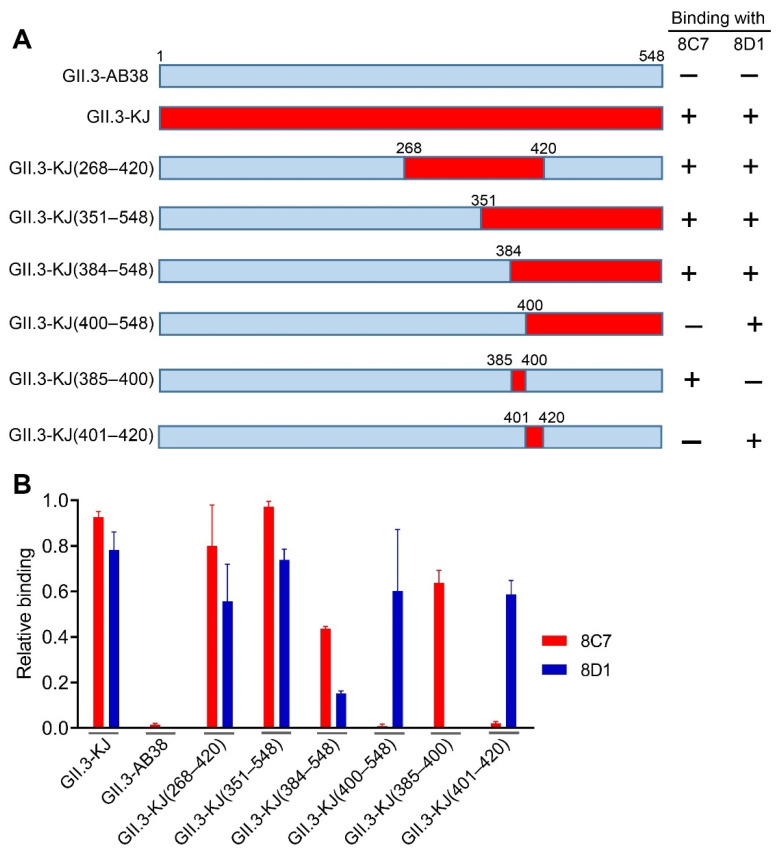
Epitope mapping of mAbs 8C7 and 8D1. (**A**) Diagram of the parental and chimeric GII.3 VLPs. The summary of the VLP-binding ELISA results is shown on the right-hand side. +, positive result; –, negative result. (**B**) Binding activities of the 8C7 and 8D1 mAbs to the indicated parental and chimeric GII.3 VLPs were tested by ELISA. For a given VLP sample, its reactivity with 8C7 or 8D1 was normalized against that with rabbit polyclonal antibody raised against GII.3 VLP, and relative binding was calculated as follows: (OD450 value obtained with mAb—OD450 value of blank well)/(OD450 value obtained with anti-GII.3 polyclonal antibody—OD450 value of blank well). Data are mean ± SEM of triplicate samples.

To roughly locate the epitopes of blockade antibodies 8C7 and 8D1, we used GII.3-AB38, which did not react with the two mAbs, as the backbone to construct a series of chimeric VLPs. Briefly, fragments of the GII.3-AB38 VP1 protein were replaced with the corresponding ones from the immunogen strain GII.3-KJ (Figure 4A). The resulting chimeric VLPs were analyzed for their reactivity with 8C7, 8D1, and the anti-GII.3 sera by ELISA. The results were summarized in Figure 4A, and representative data were shown in Figure 4B. Specifically, GII.3-KJ(268–420), which contained the exact P2 domain (amino acids 268–420) of the GII.3-KJ VP1 protein, could react with both 8C7 and 8D1, indicating that the P2 domain harbors the epitopes of the two mAbs. GII.3-KJ(351–548) and GII.3-KJ(384–548) positively reacted with 8C7 whereas GII.3-KJ(400–548) failed to do (Figure 4A,B), suggesting that the 8C7 epitope may be located within residues 384–400. Meanwhile, 8D1 mAb bound to each of the above four chimeric VLPs, inferring that the 8D1 epitope may encompass residues 401–420.

We then substituted residues 385–400 or residues 401–420 in the VP1 protein of GII.3-AB38 with the counterparts from GII.3-KJ, resulting in two additional chimeric VLPs termed GII.3-KJ(385–400) and GII.3-KJ(401–420), respectively (Figure 4A). ELISA analysis showed that 8C7 but not 8D1 could react with GII.3-KJ(385–400) (Figure 4B). Inversely, 8D1 but not 8C7 could bind GII.3-KJ(401–420) (Figure 4B). These data confirmed that 8C7 and 8D1 indeed recognize two distinct epitopes located within the residues 385–400 and 401–420 of the GII.3-KJ VP1 protein, respectively.

### 3.5. Sequence Alignment of the 8C7 and 8D1 Blockade Epitopes

**Figure 5:**
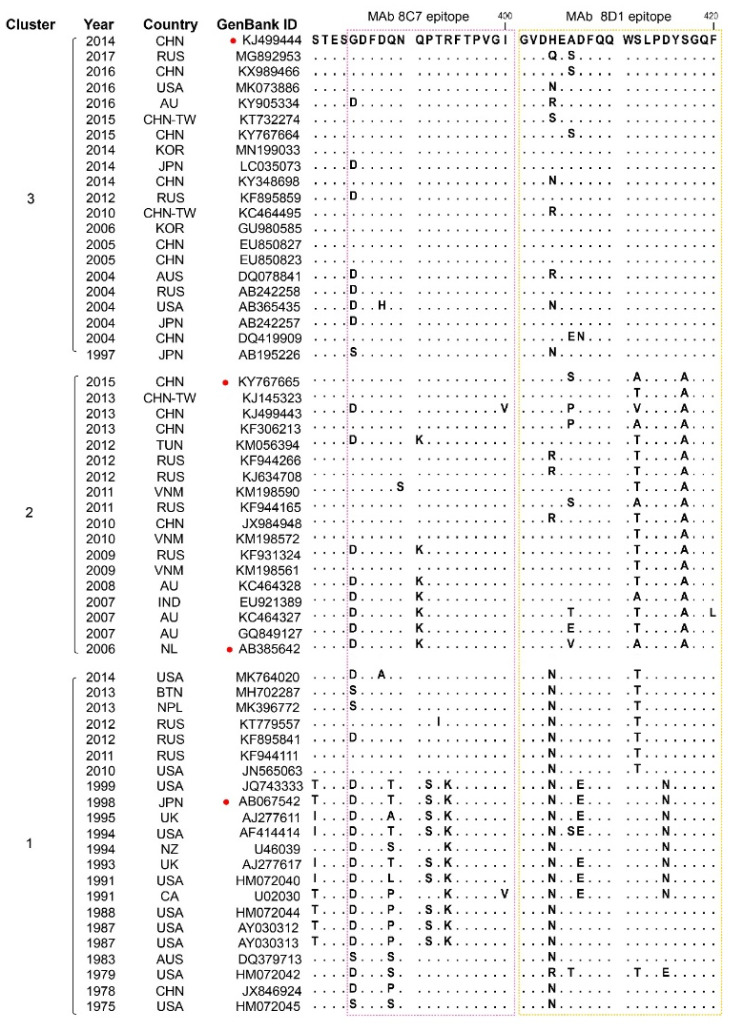
Alignment of the corresponding 8C7 and 8D1 epitope sequences from representative strains of different GII.3 clusters. Information for each strain, including isolation date (year), isolation location (country), and GenBank ID, were shown. The 8C7 and 8D1 epitope regions were boxed with dash lines. Dots represent residues identical to those of the GII.3-KJ strain. Red circle indicates the strains used in this study.

To evaluate whether the 8C7 and 8D1 epitopes are conserved, we compared the corresponding epitope sequences of representative strains from GII.3 clusters 1, 2, and 3. Sequence alignment reveals that the 8C7 epitope is highly conserved among the cluster 3 strains, less conserved among the cluster 2 strains, and the least conserved among the cluster 1 strains (Figure 5). It is worth noting that the 8C7 epitope is also found in several cluster 2 and 1 norovirus strains (KY767665, KJ145323, KF306213, KF944111, JN565063, etc.) (Figure 5). It is reasonable to infer that these strains should react with mAb 8C7. Indeed, mAb 8C7 can react with KY767665 (KY) strain-derived VLP (Figure 3). Similarly, the identity of the 8D1 epitope within cluster 3 is higher than that within clusters 2 or 1. In general, more amino acid changes in both 8C7 and 8D1 epitopes are seen in the cluster 1 and 2 strains relative to the cluster 3 strains. Interestingly, it is noted that all of the cluster 2 strains analyzed have residue replacements at positions 412 and 417 within the 8D1 epitope (Figure 5).

### 3.6. Structural Modeling of the 8C7 and 8D1 Epitopes

**Figure 6:**
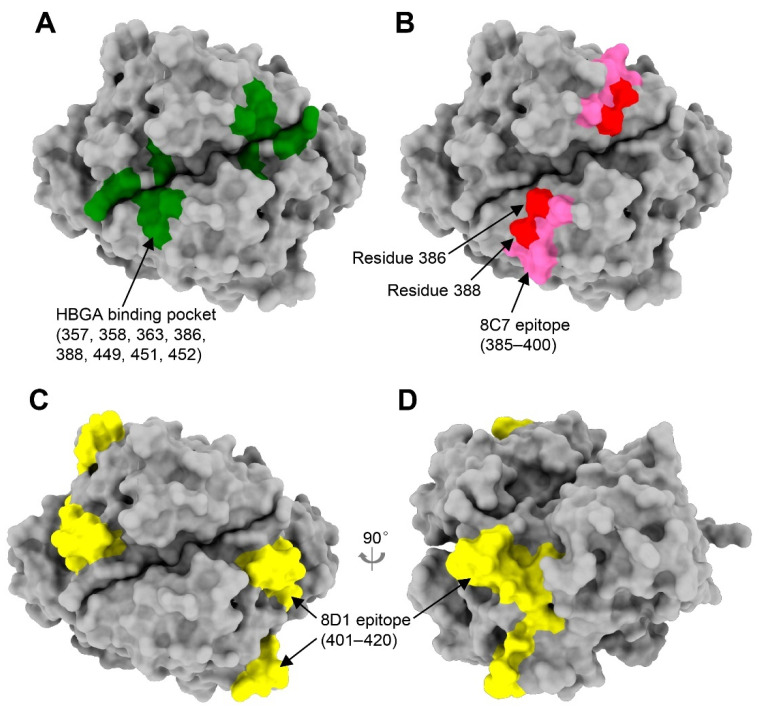
The 8C7 and 8D1 epitopes were mapped onto the surface of the GII.3 P domain dimer (strain TV-24; PDB: 6IR5). (**A**) Model of GII.3 P domain showing the HBGA binding site (green). (**B**) Location of 8C7 epitope (hot pink) on GII.3 P domain surface. Residues 386 and 388 are shown in red. (**C**,**D**) Location of 8D1 epitope (yellow) on GII.3 P domain surface. Panel (**A**–**C**), top view; panel (**D**), side view. The corresponding residues in the HBGA binding site or the mAb epitopes are included in the parentheses.

A recent structural study has defined the HBGA binding site on the GII.3 strain TV24 (GenBank code: U02030) P domain, which is shaped primarily by eight residues including T357, R358, K363, D386, D388, S449, G451, and R452 [36]. These eight residues are identical among the TV24 strain (cluster 1) and the four GII.3 strains tested here (data not shown), suggesting that GII.3 maintains a conserved HBGA binding site despite the virus undergoing steady evolution. Together these residues form the HBGA binding pocket on the surface of the viral capsid (Figure 6A). Structural modeling shows that the 8C7 epitope (residues 385–400) appears highly surface exposed and partially overlaps with the HBGA binding site (e.g., residues D386 and D388), indicating that 8C7 binding to the capsid may directly occlude the access of HBGAs (Figure 6B). The 8D1 epitope (residues 401–420) is also well exposed on the surface (Figure 6C,D). Although having no overlap with the HBGA binding site, a part of the 8D1 epitope (residues 401–405) is located immediately adjacent to the HBGA binding site in tertiary structure (Figure 6C), suggesting that 8D1 may interfere with HBGA binding through steric hindrance.

## 4. Discussion

Information on blockade antibody epitopes of specific NoV genotypes is important for understanding the antigenic landscape and evolution of these viruses [38,50,51,52]. However, prior to this study, GII.3 blockade antibody epitopes have not been located and verified due primarily to the lack of GII.3-specific blockade mAbs. Although two anti-GII.3 mAbs were isolated in previous studies, these mAbs had no HBGA-blocking activity, in agreement with their corresponding epitopes being mapped to the S domain, which plays no role in HBGA binding [53,54]. In the present study, we identified two GII.3-specific mAbs, 8C7 and 8D1, with potent blockade activity and mapped their corresponding epitopes to residues 385–400 and 401–420, respectively, within the P2 domain. The experimental definition of blockade antibody epitopes of GII.3 norovirus is a novel observation.

The 8C7 epitope comprises residues 385–400, where several previously identified GII.4 blockade epitopes reside or overlap, including the epitope D consisting of residues 391 and 393–396 [49,52,55], the 3C3G3 epitope [56], and the 10E9 epitope [57]. The 8C7 epitope is located on the surface of the GII.3 viral capsid (Figure 6B). It is noted that the 8C7 epitope overlaps with the HBGA binding site (HBS) by two residues D386 and D388 (Figure 6A,B), thus explaining the blockade activity of 8C7 mAb and also suggesting that 8C7 binding to the viral capsid may directly occlude the access of HBGAs. The 8D1 epitope defined by residues 401–420 also appears well exposed on the capsid surface (Figure 6C). Within the 8D1 epitope region, the blockade epitope E of GII.4 (comprising residues 407, 412, and 413) also resides [51]. Unlike the 8C7 epitope, the 8D1 epitope does not share common residues with the HBS. However, structural modeling shows that the 8D1 epitope (especially residues 401–409, which form a surface loop) is spatially adjacent to the HBGA binding pocket (Figure 6A,C), suggesting that 8D1 may interfere with HBGA binding via steric hindrance rather than direct occlusion. Based on the fact that multiple blockade antibody epitopes so far identified for either GII.3 or GII.4 involve the regions encompassing residues 385–400 or residues 401–420, it is likely that these regions constitute two distinct “hot spots” targeted by the immune system for induction of blockade antibodies.

Overall, the 8C7 epitope sequence is highly conserved within the GII.3 cluster 3, less well conserved in cluster 2, and the least conserved in cluster 1 (Figure 5). Similarly, the identity of the 8D1 epitope sequence is higher in cluster 3 than in clusters 1 and 2 (Figure 5). Compared to the immunogen strain GII.3-KJ (cluster 3), the strain GII.3-AB38 (cluster 2) contains two and three amino acid variations in the 8C7 and 8D1 epitopes, respectively, while the strain GII.3-AB06 (cluster 1) carries three residue changes in each of the two epitopes, explaining why these two strains failed to react with 8C7 and 8D1 mAbs (Figure 3 and Figure 5). These results also show that GII.3 has been undergoing antigenic drift. Interestingly, we observed that all strains in clusters 2 and 3 have glutamine (Q) at position 389 (Q389) located within the 8C7 epitope, whereas this residue is substituted (Q389T/P/L/S/A) in the majority of the cluster 1 strains analyzed; additionally, simultaneous residue changes at positions 412 (S412A/V/T) and 417 (S417A) within the 8D1 epitope are seen exclusively in the cluster 2 strains but not in the clusters 1 and 3 (Figure 5). These findings suggest that the residues at sites 389, 412, and 417 or their combinations may serve as signatures for individual clusters. For example, a combination of Q389, S412, and S417 could be used as the signature for cluster 3, while A417 could serve as the signature for cluster 2. It is worth pointing out that site 389 is one of the positive selection sites identified in previous evolutionary analyses [21,22,33]. In addition, site 385 in the 8C7 epitope and site 406 in the 8D1 epitope are also positively selected sites [21,22,33]. Taken together, our data show that GII.3 NoVs indeed undergo antigenic drift, likely driven by host-posed selective pressure. Such antigenic variation, together with intergenic recombination, may have contributed to the evolution of GII.3 NoVs [21,22,33].

In summary, the present study developed a set of anti-GII.3 mAbs and experimentally defined blockade antibody epitopes of GII.3 norovirus, not only enhancing our understanding of GII.3 norovirus evolution but also providing information critical for anti-GII.3 norovirus vaccine development.

## Figures and Tables

**Table 1:** A summary of all the GII.3 VLPs used in this study.

VLP	Strain	GenBank ID	Cluster ^b^
GII.3-KJ ^a^	Hu/GII.3/CUHK-NS-227/2014/CHN	KJ499444	3
GII.3-KY	Hu/GII.3/3-34/2015/HNZZ/CHN	KY767665	2
GII.3-AB38	Hu/GII.3/RotterdamP8D31/2006/NL	AB385642	2
GII.3-AB06	Hu/NoV/GII.3/SaitamaU201/1998/JPN	AB067542	1

^a^ GII.3-KJ VLP was used to produce the mAbs. ^b^ According to Saito et al. [33].

**Table 2:** Characteristics of the anti-GII.3 mAbs.

MAb	Isotype	Binding Activity to GII.3 VLP ^a^	Binding Affinity with GII.3 VLP ^b^		
			KD (nM)	Kon (1/Ms)	Kdis (1/s)
8C7	IgG1	+	<0.001	1.88 × 10^5^	<1.0 × 10^−7^
8D1	IgG1	+	<0.001	3.14 × 10^5^	<1.0 × 10^−7^
9B8	IgG1	+	0.161	4.15 × 10^5^	6.66 × 10^−5^
3A3	IgG1	+	31.0	6.1 × 10^3^	1.89 × 10^−4^

^a^ Hybridoma culture supernatants were tested for binding to GII.3 VLP by ELISA. +, OD450 > 0.5. ^b^ Binding affinities of the MAbs to GII.3 VLP were measured by BLI. Related to Figure 1.

## Data Availability

The data presented in this study are available on request from the corresponding author.
